# Genomic insights into uterine leiomyosarcoma: Unraveling *KMT2D, CREBBP, NOTCH2, TSC2, ATM*, and *GNAS* signatures

**DOI:** 10.1016/j.clinsp.2026.101043

**Published:** 2026-07-17

**Authors:** Laura Gonzalez dos Anjos, Leonardo Tomiatti da Costa, Daniela Bizinelli, Tatielly Teles Miranda, Edmund Chada Baracat, Katia Candido Carvalho

**Affiliations:** a Laboratório de Ginecologia Estrutural e Molecular (LIM 58), Disciplina de Ginecologia, Departamento de Obstetrícia e Ginecologia, Hospital das Clínicas da Faculdade de Medicina da Universidade de São Paulo (HCFMUSP), São Paulo, SP, Brazil; b Interunit Graduate Program in Bioinformatics, Instituto de Química da Universidade de São Paulo, São Paulo, SP, Brazil

**Keywords:** Leiomyosarcoma, Uterine neoplasms, Gene expression, Methylation, Point mutation

## Abstract

Uterine Leiomyosarcoma (ULMS), characterized by diagnostic and therapeutic challenges, remains poorly understood despite advancements. Previous research identified genetic mutations in ULMS samples, suggesting their role in pathogenesis. This study deepened the exploration, focusing on specific genes (*KMT2D, CREBBP, NOTCH2, ATM, TSC2, GNAS*), validating mutations, examining gene methylation, and correlating with clinical features. Thirty samples, including 15 Formalin-Fixed Paraffin-Embedded (FFPE) and 15 fresh-frozen ULMS, were collected, comprising 3 ULMS, 3 Uterine Leiomyomas (ULM), 3 Myometrial samples (MM), and 6 other Uterine Sarcomas (US) for each sample set. Two cell lines, ULM (THESCs – CRL ‒ 4003) and ULMS (SK-UT-1 ‒ HTB-114), were used for comprehensive analysis. FFPE samples underwent genomic DNA extraction using the QIAamp DNA FFPE Tissue Kit, and methylation levels were assessed using the Illumina Infinium Methylation EPIC BeadChip 850k system. Genomic DNA and RNA were extracted using the Trizol method. qRT-PCR with TaqMan assays analyzed gene expression. Mutations in *CREBBP, NOTCH2*, and *GNAS* were sequenced using the Sanger method. Results indicated downregulation in *CREBBP, GNAS*, and *NOTCH2* genes in ULMS. Sequencing revealed specific mutations (e.g., c.4063G>A in *CREBBP*, T>A at position 78 in *NOTCH2*). A pronounced reduction in CpG region methylation suggested a unique epigenetic alteration in ULMS. Methylation with clinical outcomes identified *ATM* as a potential independent prognostic biomarker. In conclusion, this study provides a comprehensive view of ULMS molecular complexity, potentially influencing clinical practices.

## Highlights

• Integrative multi-omic analysis of Uterine Leiomyosarcoma (ULMS), combining mutation validation, gene expression profiling, and DNA methylation analysis of six key genes.
• The results reveal coordinated downregulation and hypomethylation of CREBBP, GNAS, and NOTCH2, and identify ATM promoter hypermethylation as an independent prognostic marker associated with improved survival.
• Novel molecular features that may support the future use of prognostic biomarkers and targeted therapies.

## Introduction

Uterine Leiomyosarcoma (ULMS), which accounts for approximately 70% of Uterine Sarcomas (US), is a rare but highly aggressive malignancy that predominantly affects women between 50- and 60-years of age. Although uncommon, ULMS represents 3%–9% of uterine cancers and is associated with a significantly poorer prognosis compared with more frequent epithelial uterine tumors.^1^^,^^2^

The clinical and biological complexity of ULMS extends beyond its rarity. Tumor development involves a combination of genetic and epigenetic alterations, including somatic mutations, histone modifications, DNA methylation changes, and dysregulation of microRNA expression.^3^^,^^4^ Genetic mutations and DNA methylation can modulate gene expression and cellular behavior, though their impact depends on a complex interplay of individual genetic, epigenetic, and environmental factors. This heterogeneity exists both between individuals and among different cell populations within the same tumor, underscoring the importance of integrative molecular approaches in cancer research.^5^

Although evidence increasingly supports the role of these mechanisms, integrated analyses simultaneously evaluating genetic alterations, epigenetic changes, and their functional consequences in ULMS remain limited. In this context, our group recently performed a comprehensive genetic analysis of 23 FFPE uterine sarcoma and carcinosarcoma samples, identifying recurrent pathogenic mutations in TP53, ATM, and PIK3CA.^6^ In uterine leiomyosarcoma samples, additional pathogenic mutations were observed in KMT2D, CREBBP, NOTCH2, TSC2, ATM, and GNAS, genes involved in key regulatory pathways related to transcriptional control, cell growth, DNA damage response, and cellular homeostasis.^6^^,^^7^

Despite growing recognition of the roles of genetic and epigenetic mechanisms in tumor biology, their combined contribution to ULMS pathogenesis remains insufficiently characterized. The rarity of ULMS and the limited number of studies focusing on its epigenetic landscape have likely hindered the identification of robust molecular targets and the development of effective targeted therapies.^4^, ^5^, ^6^, ^7^, ^8^ Therefore, this study aimed to characterize somatic mutations and DNA methylation patterns in ULMS and to evaluate their associations with gene expression profiles and clinicopathological features. As secondary objectives, the authors assessed the potential impact of these molecular alterations on patient outcomes, contributing to a more integrated understanding of ULMS biology and supporting future advances in personalized therapeutic strategies.

## Materials and methods

### Cell cultures and human sample selection

The ULM and ULMS cell lines (ATCC CRL-4003 and SK-UT-1 HTB-114) were obtained from the American Type Culture Collection (ATCC). Cells were cultured in specific media, incubated at 37 °C with 5% CO_2_ until reaching a density of 5 × 10^6^ cells, then harvested, washed, and stored at −20 °C. DNA isolation was done using the TRIzol method (Thermo Fisher, USA), and authentication was performed via sequencing with the GenePrint 10 System kit (Promega, USA), referencing loci in the ATCC database.

A total of 30 samples were analyzed, comprising 15 FFPE and 15 fresh-frozen tissues. All FFPE samples consisted exclusively of ULMS cases obtained from the Instituto do Câncer do Estado de São Paulo (ICESP, São Paulo, Brazil). The fresh-frozen set included 3 ULMS, 6 other Uterine Sarcomas (US), 3 Uterine Leiomyomas (ULM), and 3 Myometrium (MM) samples. All ULMS and other sarcoma samples within the fresh-frozen group were sourced from ICESP and the Instituto Brasileiro de Controle do Câncer (IBCC), whereas all ULM and MM fresh-frozen samples originated from the Hospital das Clínicas, Faculty of Medicine, University of São Paulo (HCFMUSP, São Paulo, Brazil). Human tissues were selected based on strict diagnostic and quality criteria. ULMS, ULM, and US samples were included only after confirmation by an experienced pathologist following WHO diagnostic standards. MM controls were obtained from hysterectomies performed for benign conditions and were included only when no malignancy, inflammation, or infection was identified. Exclusion criteria included inconclusive diagnoses, insufficient or degraded tissue, prior neoadjuvant therapy, concurrent gynecologic malignancies, or incomplete clinical information. After applying these criteria, all 30 samples met the requirements for molecular analysis. Ethical approval was obtained from the Institutional Research Ethics Committees (registration #3.524.384, #3.552.748, #NP 1497/19).

Fresh-frozen samples were used exclusively for gene expression and Sanger sequencing analyses due to their superior RNA and DNA integrity, whereas FFPE samples were used exclusively for DNA methylation profiling. No assay combined FFPE and fresh-frozen tissues, and no paired samples were included.

Detailed metadata for all samples, including preservation method, molecular assay performed, tumor category, institution of origin, and collection years, are provided in Supplementary Table 1 and Supplementary Table 2. A summary of sample distribution across molecular assays is provided in Supplementary Table 3.

### DNA and RNA isolation

DNA from FFPE tissues was isolated using the QIAamp DNA FFPE Tissue Kit (Qiagen) following all the manufacturer's steps. The protocol included xylene-based deparaffinization, ethanol washes, heat-induced reversal of formalin crosslinking, proteinase-K digestion, and DNA purification. Only DNA samples with A260/280 ratios of 1.8–2.0 and detection p-values < 0.2 were considered suitable for methylation analysis. Fresh-frozen tissues were processed with the AllPrep DNA/RNA Mini Kit (Qiagen), allowing simultaneous purification of RNA and DNA. RNA underwent an additional cleanup step with RNeasy MinElute (Qiagen) to eliminate genomic DNA contamination. RNA integrity was confirmed by A260/280 ratios and the presence of clear 18S and 28S rRNA bands.

### Quantitative reverse transcription PCR (qRT-PCR) analysis

RNA samples were used for gene expression assays of KMT2D, CREBBP, NOTCH2, ATM, TSC2, and GNAS. cDNA synthesis used the High-Capacity cDNA Reverse Transcription Kit (Thermo Fisher Scientific, USA). Gene expression assays were conducted using TaqMan probes (Thermo Fisher Scientific, USA). Specific regions for each gene were covered (listed in the full text). Beta-2-Microglobulin (B2M) was the endogenous control. Reactions were performed in duplicates, using 10 µL of 2× TaqMan Universal PCR Master Mix, 1 µL of TaqMan assay, and 9 µL of cDNA in RNase-free ultrapure water (1:10). Temperature cycling was done using the 7500 Real-Time PCR System (Thermo Fisher Scientific, USA), and data were analyzed with the 2^−ΔΔCt^ method using 7500 v.2.3 software.

All primer sequences, amplicon sizes, and cycling conditions used for qPCR were standardized and applied uniformly across all samples to ensure methodological reproducibility.

### DNA methylation assay

Raw IDAT files were imported into R (v.3.6) using the minfi package. Background correction and quality assessment were performed using array control probes. Probes were removed if they had a detection p-value > 0.01, bead count 〈 3 in 〉 5% of samples, cross-reactive or multi-hit alignment profiles, SNPs at the CpG or single-base extension sites, or if located on sex chromosomes. Sample-level exclusion was based on a detection p-value > 0.2. Normalization was performed with BMIQ to reduce Type I/II probe bias. Batch correction was applied using ComBat from the sva package.

External DNA methylation data (n = 10 myometrium samples from GSE120854) were only available as processed β-values, preventing joint raw-level preprocessing with the internal cohort. Thus, internal samples were fully processed from IDAT files (QC, probe filtering, and BMIQ normalization), and the external β-values were incorporated afterward. To harmonize datasets, ComBat (sva package) was applied to the combined β-value matrix using *dataset origin* (internal vs. GEO) as the batch variable, while including the biological condition (ULMS vs. MM) in the model matrix to avoid overcorrection. The effectiveness of batch removal was evaluated using PCA/MDS and hierarchical clustering before and after ComBat. Differentially Methylated Positions (DMPs) were defined as those with FDR-adjusted p-value < 0.05 and |Δβ| ≥ 0.20. Differentially Methylated Regions (DMRs) were identified using DMRcate, requiring ≥ 2 CpGs within 500 bp and FDR < 0.05. These results are shown in Supplementary Figure 1 and demonstrate a substantial reduction in dataset-origin bias.

### Quality control and bioinformatics analysis

Quality control was obtained using the minfi package (v.1.48.0) from Bioconductor for R (v.4.3.2). Samples with p-value > 0.2 were removed, and probes mapped to the X and Y chromosomes, cross-reactive probes, and those with low reproducibility or missing data in > 10% of samples were filtered out. Data normalization was performed using the BMIQ method.^9^^,^^10^ The sva package (v.3.50.0) was used to remove batch effects.^11^ External normal samples (MM, n = 10) from the Gene Expression Omnibus (GEO) repository (GSE120854) were incorporated. Differentially Methylated Positions (DMPs) were identified with an FDR-adjusted p-value < 0.05 and |Δβ| ≥ 0.2. Differentially Methylated Regions (DMRs) were identified using DMRcate (v.2.10.0) (FDR < 0.05, ≥ 2 CpGs within 500 base pairs).^12^

All NOTCH2 probes were excluded automatically during QC because they failed standard thresholds (detection p-value > 0.01 or bead count 〈 3 in 〉 5% of samples). These criteria were applied globally to all probes, and therefore NOTCH2 did not proceed with downstream analysis.

### Sanger sequencing for mutation identification

Conventional PCR amplified regions for sequencing, with primer sequences designed for identified mutations. PCR reactions were purified with the Qiaquick PCR Purification kit (Qiagen, Germany), quantified with the NanoDrop 2000 (Thermo Fisher Scientific, USA), and sequenced using the Big Dye Terminator protocol, following the manufacturer’s instructions, on the 3500 Genetic Analyzer (Thermo Fisher Scientific, USA). Data collection was done with Data Collection software, and electropherograms were analyzed with Chromas 2.6.6 (Technelysium, Australia), Minor Variant Finder (Thermo Fisher Scientific, USA), and SnapGene Viewer 7.0.1 (Dotmatics, UK). Sequence alignment was done with SnapGene and the Sequence Alignment tool (VectorBuilder, USA).

### Statistical analysis

Statistical analyses were conducted using GraphPad Prism v.5.0 and SPSS v.23. Data reliability was assessed using the Shapiro-Wilk test. Parametric tests, such as *t*-tests or ANOVA, were used for normally distributed variables, while non-parametric tests like Mann-Whitney *U* or Kruskal-Wallis were used otherwise. The Mann-Whitney *U test* evaluated methylation status in ULMS and MM and gene expression differences between ULM and ULMS. Gene expression in cell cultures was analyzed with the Kruskal-Wallis test and Dunn's post-test. Overall Survival (OS) curves were generated using the Kaplan-Meier method, with significance assessed by the log-rank test. Cox regression analyzed the impact of independent variables on survival. Survival was based on β-values (up to and ≥ median), and survival rates were calculated in months from surgery to death or last follow-up. Disease-Free Survival (DFS) was calculated from the time of surgery until recurrence. Statistical significance was set at p < 0.05. A total of 11 events were observed for overall survival and 12 for disease-free survival. Hazard ratios with corresponding 95% confidence intervals were calculated. Given the limited number of events relative to the number of variables included in the models, Cox regression estimates should be interpreted with caution.

Raw data were generated at the Laboratory of Structural and Molecular Gynecology (LIM-58), Faculdade de Medicina, Universidade de São Paulo, and Neogen do Brasil. Derived data are available from the corresponding author upon request.

## Results

**Fig. 1:**
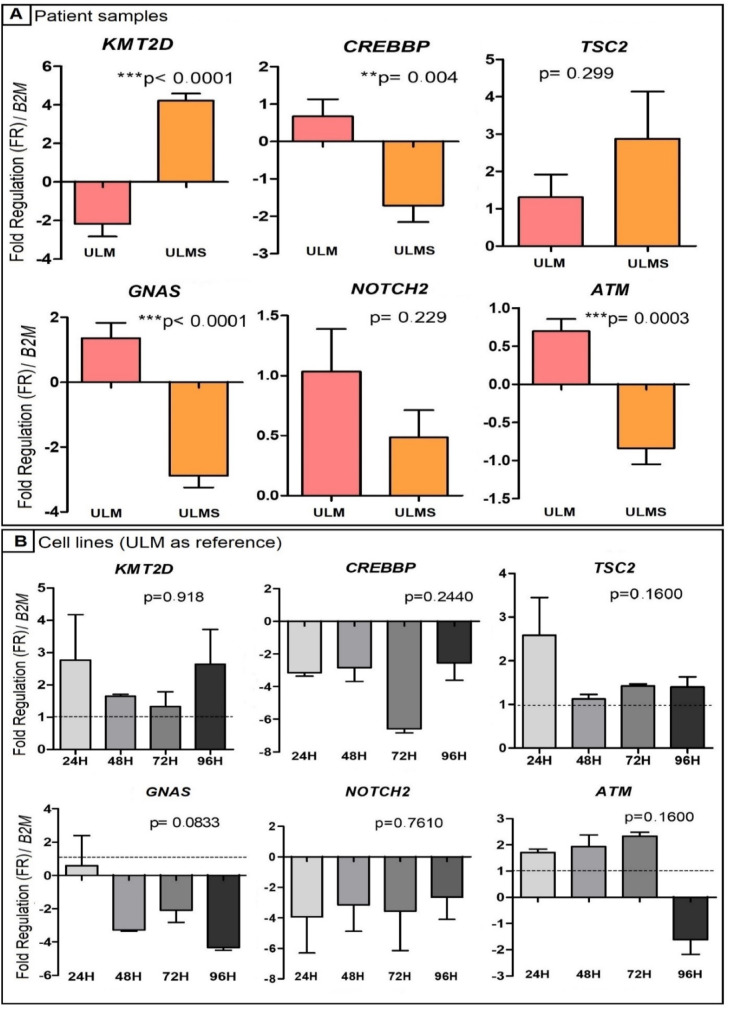
Expression of *KMT2D, CREBBP, TSC2, GNAS, NOTCH2*, and *ATM* genes in ULMS. (A) Graphs representing the gene expression in samples from patients with ULMS and ULM compared to MM. (B) Graphs representing the gene expression in ULMS cell culture compared to ULM cells at 24 h, 48 h, 72 h, and 96 h time points.

The authors collected clinical and pathological data from medical records, including age, postmenopausal bleeding, main complaints, parity, pathology, treatment, recurrence/metastasis, metastasis location, and status (Table 1). Case selection was based on available FFPE samples to ensure thorough methylation analysis. Patient ages ranged from 38- to 85-years (mean 57.8 ± 15.6), most presented postmenopausal bleeding, and over 80% experienced recurrence or metastasis (Table 1). qRT-PCR analysis revealed significant differences in gene expression, particularly for CREBBP, GNAS, and NOTCH2 in ULMS. CREBBP expression was significantly lower in ULMS compared to ULM (p = 0.004), with GNAS showing a more pronounced decrease (p < 0.0001) (Fig. 1A). In cell cultures, CREBBP decreased after 72-hours (FR = −6.57), GNAS after 96-hours (FR = −4.31), and NOTCH2 after 24-hours (FR = −3.93) (Fig. 1B). Comparative analysis between patient samples and cell lines for KMT2D, CREBBP, TSC2, GNAS, NOTCH2, and ATM showed consistent expression patterns despite differences in fold change values.

**Table 1:** Clinicopathological characteristics of patients diagnosed with ULMS selected for methylation analysis (n = 15).

Variables	Categories	ULMS n (%)
Age	≥ 50-years	9 (60)
	< 50-years	6 (40)
Postmenopausal bleeding	Yes	9 (60)
	No	5 (33)
	N.A.	1 (7)
Main Complaint	Without complaint	1 (7)
	Abnormal bleeding	8 (53)
	Abdominal pain	5 (33)
	N.A.	1 (7)
Parity	Nulliparous	3 (20)
	Multiparous	8 (53)
	N.A.	4 (27)
Underlying pathology	Yes	8 (53)
	No	4 (27)
	N.A.	3 (20)
Adjuvant treatment	CT	4 (27)
	RT	5 (33)
	CT + RT	3 (20)
	No	3 (20)
Recurrence / Metastasis	Yes	13 (87)
	No	2 (13)
Metastasis location	Lungs	7 (47)
	Liver	1 (7)
	Lungs + Liver	3 (20)
	Others	2 (13)
	N.A.	2 (13)
Status	Alive	2 (13)
	Death	12 (80)
	Loss of follow-up	1 (7)

In this comprehensive analysis, the authors observed a pronounced upregulation of gene expression, notably in the KMT2D and TSC2 genes, across both uterine ULMS patient samples and cell lines. Further investigation into gene expression attenuation led us to prioritize CREBBP, GNAS, and NOTCH2, focusing on validating previously identified mutations through Next-Generation Sequencing (NGS). This selection was based on the significance of reduced expression in oncogenic contexts, including tumor suppression, silent mutations impacting gene regulation, signaling pathways, clinical relevance, targeted therapies, and the intrinsic heterogeneity of cancer.

It is important to emphasize the deliberate exclusion of initial nucleotides during genomic sequencing in the sequence alignment process to mitigate biases from low-quality data. This strategy improves data interpretation clarity, ensuring accurate identification of genetic variations and maintaining the integrity of findings.

**Fig. 2:**
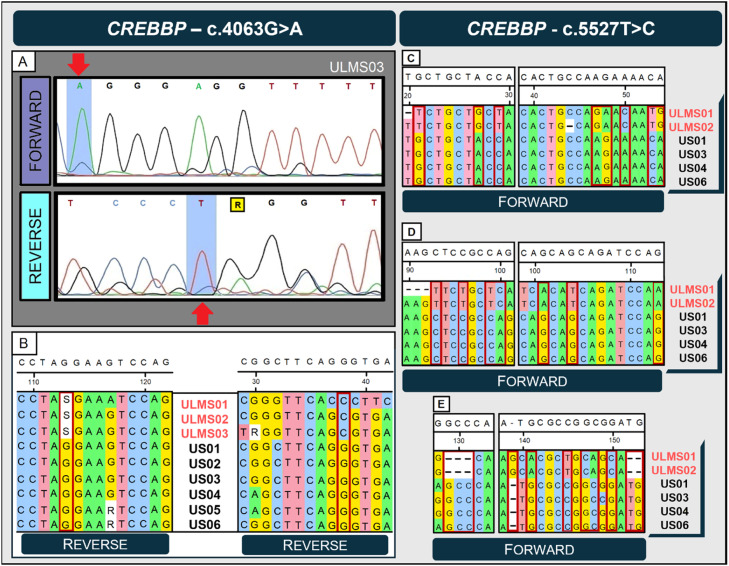
Analysis of *CREBBP* mutations in ULMS. (A) c.4063G>A mutation in ULMS03 displayed homologous manifestations in both forward (G>A) and reverse (C>T) strands. (B) Unique alterations in the reverse strand, such as a G>C substitution at position 38 and the presence of the degenerate base S at position 113, set ULMS apart from other types of US. (C) ULMS01 and ULMS02 exhibited the highest rates of base substitutions, featuring specific mutations like G>T at position 21, A>G at positions 27, 47, and 54, and A>C at position 50. (D) Additional transitions and transversions observed in ULMS samples included C>T at positions 93, 96, and 99, G>A at positions 101 and 113, and G>C at position 150. (E) Unique deletion events at positions 129, 130, 131, 152, and 153, with the presence of a G at position 139 among deletions, were identified. ULMS01 and ULMS02 showcased distinct genetic patterns, contributing to the understanding of CREBBP mutations in ULMS.

**Fig. 3:**
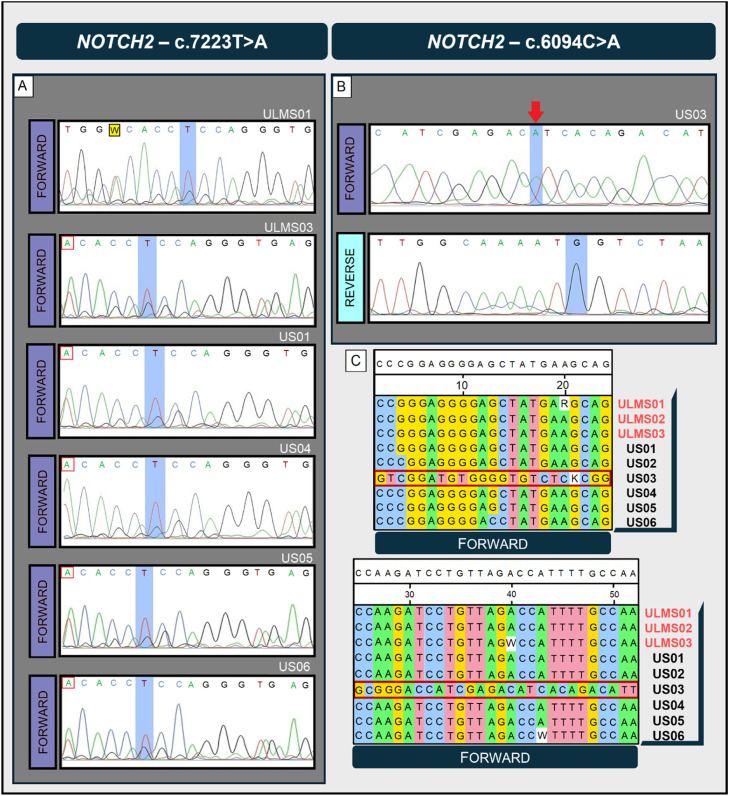
Analysis of *NOTCH2* mutations in ULMS. (A) Despite the absence of the c.7223T>A mutation in the *NOTCH2* gene, the comprehensive analysis revealed intriguing characteristics in this genomic region. At position 78, a unique transversion (T>A) was identified in five samples, potentially serving as a genetic marker influencing distinct tumor characteristics. (B) Exclusive identification of the c.6094C>A mutation in *NOTCH2* was observed in the forward sequence of sample SU03. (C) Significant alterations in bases 2 to 52 set SU03 apart from other samples. Distinctive genetic factors or exceptional conditions linked to this sample underscore a remarkable contrast from the remaining analyzed samples, emphasizing its unique profile.

**Fig. 4:**
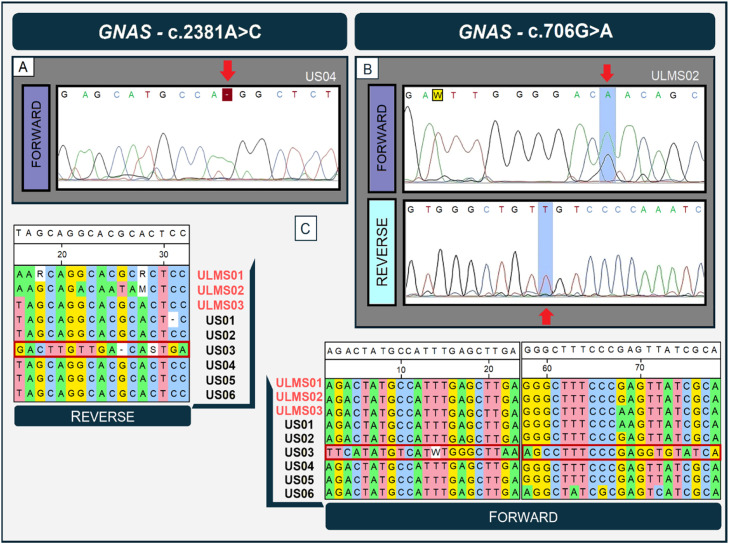
Analysis of GNAS mutations in ULMS. (A) The c.2381A>C mutation in the *GNAS* gene was absent, but in sample US04, a deletion in the forward sequence at the expected substitution position was detected. (B) The persistence of the c.706G>A mutation in ULMS02 in both forward and reverse sequences. (C) Sample US03 stands out as the most altered among others, exhibiting various alterations in the forward and reverse sequences. This unique genetic signature in US03 suggests a distinctive molecular profile.

The NGS analysis identified a c.4063G>A missense mutation in CREBBP in ULMS03, with additional base substitutions and insertions/deletions in ULMS01 and ULMS02 distinguishing them from other uterine sarcomas (Fig. 2A‒E). NOTCH2 displayed multiple substitutions and gaps, including a T>A transversion at position 78 in five samples (Fig. 3A‒C). GNAS analysis revealed deletions at positions corresponding to c.2381A>C in US04 and the persistence of c.706G>A in ULMS02 (Fig. 4A‒C).

After analyzing genetic alterations in KMT2D, CREBBP, NOTCH2, ATM, TSC2, and GNAS, the authors focused on DNA methylation, a key regulatory mechanism influencing gene expression. Although not all mutations were validated, methylation profiling provided additional insights. Gene expression changes were associated with both genetic mutations and methylation alterations. To contextualize these results, methylation levels were compared with MM samples from the GEO database, and probes in the NOTCH2 region were excluded due to potential degradation in FFPE samples.

**Fig. 5:**
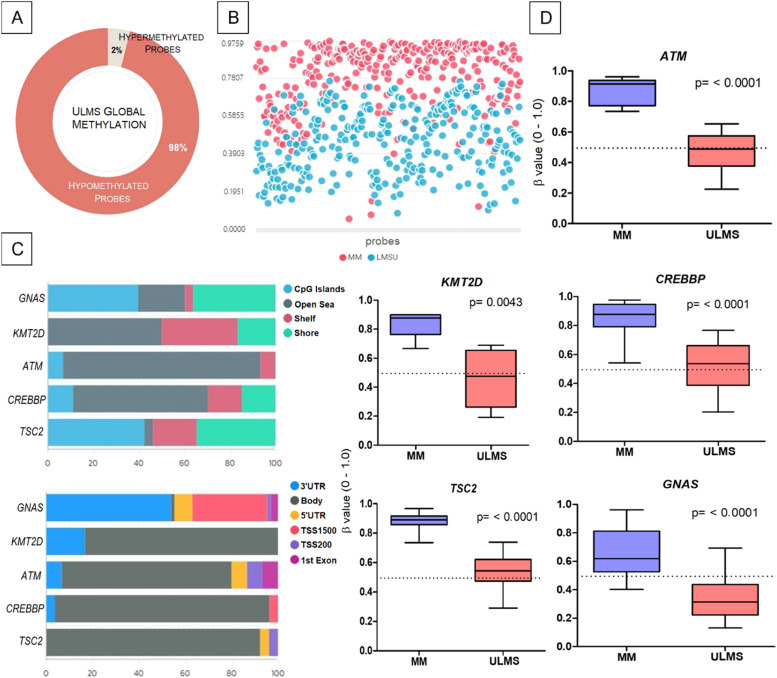
Detailed methylation profile of *GNAS, KMT2D, ATM, CREBBP*, and *TSC2* genes in ULMS. (A) Illustration of the pronounced hypomethylation trend in ULMS, covering approximately 98% of the analyzed probes, depicted by the donut chart. (B) Scatter plot representing the 132 Differentially Methylated Probes (DMPs). (C) Stacked bar chart illustrating the detailed distribution of CpG probes and the specific genomic distribution for GNAS, KMT2D, ATM, CREBBP, and TSC2 genes. (D) Significant differences in β-values in *ATM, KMT2D, CREBBP, TSC2* e *GNAS* between ULMS and MM.

Methylation analysis of KMT2D, CREBBP, ATM, TSC2, and GNAS showed consistent hypomethylation in ULMS across 98% of probes (Fig. 5A). A total of 132 differentially methylated probes were identified (Fig. 5B‒C). Significant differences were observed for KMT2D (p = 0.004), CREBBP (p < 0.001), GNAS (p < 0.001), ATM (p < 0.001), and TSC2 (p < 0.001), with Δβ-values ranging from −0.60 to 0.34 (Fig. 5D). Gene body and 3′UTR regions showed the majority of probes, suggesting specific loci for potential epigenetic regulation. NOTCH2 probes did not pass QC and, therefore, were not included in the methylation results.

**Fig. 6:**
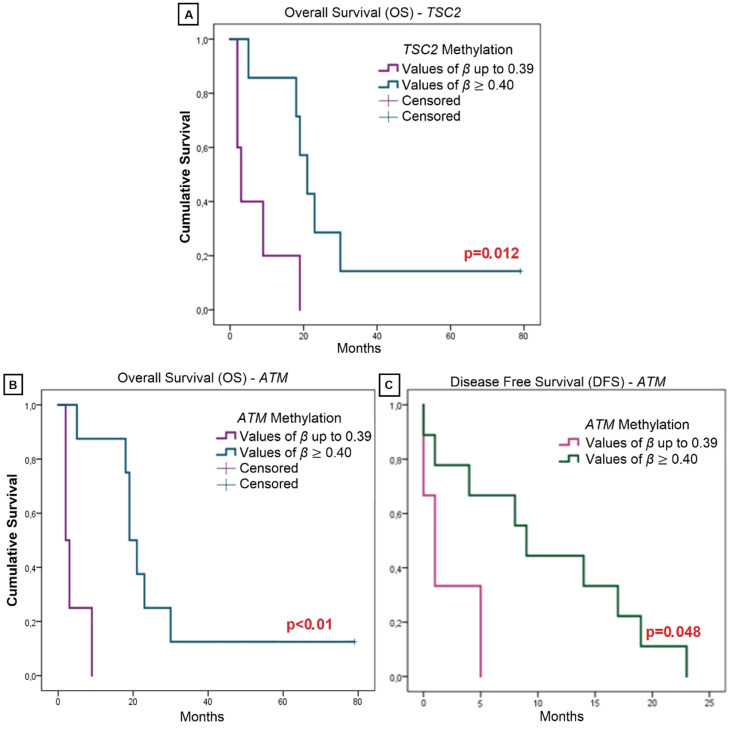
Analysis of OS and DFS concerning methylation patterns. (A) Significant differences were observed in OS between the groups with *TSC2* β-values up to 0.39 and equal to or greater than 0.40 (p = 0.01). (B) Significant differences were observed in OS between the groups with *ATM* β-values up to 0.39 and equal to or greater than 0.40 (p < 0.01). (C) Significant differences were observed in DFS between the groups with *ATM* β-values up to 0.39 and equal to or greater than 0.40 (p = 0.04).

The present study included the analysis of Overall Survival (OS) and Disease-Free Survival (DFS) concerning methylation patterns in specific genes. Samples were divided into two distinct groups: the first group with β-values up to 0.39, indicating lower levels of methylation, and the second group with β-values equal to or greater than 0.40, indicating more significant levels of methylation. These values were defined based on the median β-values of the patients' probes. The present results indicated that higher ATM and TSC2 methylation were associated with improved OS and DFS (Fig. 6A‒C). Patients with higher ATM methylation (n = 8) had longer OS (26.7 ± 7.3 months) compared to those with lower methylation (n = 4, 4.0 ± 1.68 months), and longer DFS (14.4 ± 9.5 vs. 1.25 ± 1.25 months). For TSC2, patients with higher methylation (n = 7) had OS of 27.8 ± 8.3 months versus 7.0 ± 3.3 months for lower methylation.

A univariate analysis showed that metastases and underlying pathology were the only factors significantly impacting OS (p = 0.01 and p = 0.04). No other clinical data were statistically significant. Following this, a multivariate analysis explored the relationship between methylation and clinical outcomes, particularly in the presence of metastasis and pathology. ATM methylation was significantly associated with both OS and DFS, with a reduced risk of death (HR = 0.293, p = 0.011) and a 63.5% decrease in disease recurrence risk (HR = 0.365, p = 0.016).

ATM hypermethylation is highlighted as a promising prognostic marker, correlating with better clinical outcomes. No association was found for methylation and underlying pathology in OS or DFS. KMT2D, CREBBP, GNAS, and TSC2 were inconclusive due to sample limitations. Despite this, trends in GNAS and CREBBP methylation suggested a possible reduction in adverse event risk, while KMT2D and TSC2 showed no significant associations, indicating the need for further study. In conclusion, HR estimates offer valuable insights, despite the lack of statistical significance in these cases.

Given the limited sample size, survival analyses should be interpreted cautiously, as Cox proportional hazards models may be unstable under these conditions. To complement the regression results, Kaplan-Meier curves illustrating OS and DFS for ATM and TSC2 methylation groups have been included (Fig. 6), providing a visual representation of survival differences.

## Discussion

Investigating the molecules involved in ULMS development is complex but essential for understanding its biology and improving clinical practice. The present study faces challenges, particularly due to the limited number of samples. qRT-PCR analysis revealed decreased expression of CREBBP, GNAS, and NOTCH2 genes in ULMS, suggesting that restoring their expression could potentially reverse pathological effects and contribute to the development of new therapeutic strategies.^13^

Decreased gene expression, often resulting from mutations, significantly affects genetic processes.^14^ CREBBP mutations are common in several cancers, including lymphomas, leukemia, lung, and breast cancers, serving as important genetic markers.^15^ CREBBP regulates transcriptional activation and lysine acetyltransferase functions, playing a key role in tumorigenesis.^16^ This study identified the c.4063G>A mutation in CREBBP in one of three ULMS samples, highlighting the heterogeneity of ULMS. This genetic diversity emphasizes the need for comprehensive approaches to understand ULMS variation.^6^

Further analysis of the c.5527T>C mutation in CREBBP found no evidence in these samples; however, larger studies may reveal its presence. Genetic differences between ULMS and other uterine sarcomas are crucial for identifying ULMS-specific markers. TP53 mutations and alterations in CDKN2A, ATRX, and DAXX are commonly observed in ULMS and contribute to tumor progression.^17^^,^^18^ Additionally, microRNA expression and chromosomal changes help define ULMS genetic signatures.^19^^,^^20^

NOTCH2 mutations, similar to those in CREBBP, can promote tumor progression, although the c.7223T>A mutation remains unconfirmed in ULMS samples. Further studies are required for definitive validation. Mutational alterations, including gaps and frameshift duplications, impact tumorigenesis and protein synthesis.^21^^,^^22^ A specific T>A mutation at position 78 was found in five samples, suggesting a potential ULMS-specific genetic marker. ULMS01, however, lacked this mutation, further highlighting the genetic heterogeneity of ULMS.^23^

Sample US03 exhibited more pronounced genetic alterations, suggesting increased genomic instability and mutation accumulation. Its unique genetic profile, including NOTCH2 mutations, underscores the importance of comparing different uterine sarcoma types to better understand ULMS.^23^ GNAS mutations, observed across various cancers, interfere with signaling pathways and contribute to tumor progression.^24^^,^^25^ The authors identified a c.2381A>C mutation in GNAS in sample US04, indicating potential dysregulation in gene expression,^26^ and a c.706G>A mutation in another ULMS sample.^27^

Epigenetic regulation is crucial for gene expression and genomic stability.^28^^,^^29^ Research on ULMS methylation patterns is still emerging, with studies showing methylation variations linked to tumor characteristics.^4^^,^^10^ The present findings align with previous reports of DNA hypomethylation in ULMS,^30^^,^^31^ which reported significant methylation differences between MM and ULM, reinforcing the role of methylation in ULMS biology.

DMP distribution in genes such as KMT2D, ATM, and CREBBP within open sea regions indicates complex epigenetic interactions in ULMS.^32^ Conversely, GNAS and TSC2 show DMP concentration in CpG islands, highlighting gene-specific methylation patterns.^10^^,^^33^ DMPs are mainly concentrated in gene body regions, affecting transcription and RNA processing, with GNAS showing high DMP levels in the 3′UTR, which is critical for post-transcriptional regulation.^34^

Combining epigenetic profiling with clinical data provides insights into tumor invasiveness and may guide the development of targeted therapies for ULMS. Statistical analysis revealed correlations between TSC2 methylation levels and improved overall survival, challenging the expected association between hypermethylation and gene underexpression.^34^^,^^35^ Although global CpG hypomethylation is often associated with increased transcription, the relationship between DNA methylation and gene expression is highly context-dependent.^33^^,^^34^ In the present study, methylation profiling was performed using the Illumina EPIC array, which interrogates CpG sites across multiple genomic regions and is not restricted to promoter regions. Therefore, the observed methylation patterns may not directly reflect promoter-associated transcriptional regulation. In addition, differentially methylated probes were identified in multiple genomic contexts, including gene body regions. Furthermore, analyses were conducted using tumor samples containing heterogeneous cell populations, which may influence both methylation and gene expression profiles. This cellular heterogeneity may contribute to the observed relationship between methylation status and gene expression. Increased ATM methylation also correlated with longer survival, underscoring its potential as a prognostic biomarker.^32^ Future research on ATM alterations could inform targeted treatments and enhance responses to immunotherapy.^33^^,^^34^

In summary, genetic and epigenetic analyses of ULMS reveal distinct mutation and methylation patterns, challenging traditional expectations in cancer research. These findings suggest that ATM methylation and other molecular changes could serve as prognostic markers and inform targeted therapies for ULMS.

Given the rarity of ULMS and the limited availability of high-quality tissue, the presentstudy comprises a relatively small number of cases, which inherently reduces the statistical power of some analyses and may affect the robustness of specific associations. Therefore, these findings should be considered exploratory. Additionally, functional validation and protein-level assessments could not be performed due to sample constraints, limiting direct evaluation of the biological consequences of the methylation and transcriptional alterations identified. Another limitation relates to the absence of age-matched controls, as well as the fact that age was not included as a covariate in the DMP/DMR analyses. In addition, the methylation cohort consisted exclusively of tumor samples, which limits direct comparisons with matched normal tissues. Despite these constraints, the convergence of molecular layers revealed coherent patterns and highlighted candidate genes, particularly ATM, that may play relevant roles in ULMS biology. Altogether, these findings provide an initial framework for future studies incorporating proteomic assessment, mechanistic experiments, and larger multi-omic cohorts to expand and validate the pathways and biomarkers suggested in this work.

## Conclusion

This study integrated gene expression, targeted sequencing, and DNA methylation data to explore molecular alterations in ULMS. The authors observed a predominantly hypomethylated profile and gene-specific alterations across KMT2D, CREBBP, GNAS, ATM, and TSC2, with ATM methylation showing a noteworthy association with clinical outcomes. Although the cohort size and reliance on FFPE material represent inherent limitations, these results contribute to the current understanding of ULMS and provide a basis for future studies aimed at validating these findings in larger cohorts and clarifying their potential clinical relevance.

## Statement of implication

Uterine leiomyosarcoma is an aggressive malignancy characterized by significant genomic instability. The present findings highlight the genetic mutations and DNA methylation alterations, specifically ATM methylation, as potential prognostic biomarkers. These alterations may also contribute to the development of targeted therapeutic strategies and enhance clinical management.

## Funding

Fundação de Amparo à Pesquisa do Estado de São Paulo – FAPESP (Process number: 2019/01109–2).

## Data availability

The datasets generated and/or analyzed during the current study are available from the corresponding author upon reasonable request.

## Declaration of competing interest

The authors declare no conflicts of interest.

## Appendix Supplementary materials

Image, application 1
