# Supplementary material for: Genomic insights into uterine leiomyosarcoma: Unraveling KMT2D, CREBBP, NOTCH2, TSC2, ATM, and GNAS signatures
Source: Clinics (Sao Paulo). 2026 Jul 17;81:101043. doi: 10.1016/j.clinsp.2026.101043 (PMC13401000; doi:10.1016/j.clinsp.2026.101043)

**CLINICS-D-25-01396**

**Supplementary Material**

**Supplementary Table 1** Fresh-Frozen Samples (n = 15). Samples used for gene expression and/or Sanger sequencing analysis.

| **Sample ID** | **Assay Performed** | **Tumor Category** | **Institution** | **Collection Years** | **Storage Duration** |
| --- | --- | --- | --- | --- | --- |
| ULMS_01 | Expression + Sanger | ULMS | ICESP/ IBCC | 2012–2020 | 4–12 years |
| ULMS_02 |  |  |  |  |  |
| ULMS_03 |  |  |  |  |  |
| US_01 | Gene Expression + Sanger | Other Types of Uterine Sarcoma | ICESP/ IBCC | 2012–2020 | 4–12 years |
| US_02 |  |  |  |  |  |
| US_03 |  |  |  |  |  |
| US_04 |  |  |  |  |  |
| US_05 |  |  |  |  |  |
| US_06 |  |  |  |  |  |
| ULM_01 | Gene Expression | Leiomyoma | HC-FMUSP | 2012–2020 | 4–12 years |
| ULM_02 |  |  |  |  |  |
| ULM_03 |  |  |  |  |  |
| MM_01 | Gene Expression | Myometrium | HC-FMUSP | 2012–2020 | 4–12 years |
| MM_02 |  |  |  |  |  |
| MM_03 |  |  |  |  |  |

**Supplementary Table 2** FFPE Samples (n = 15). Samples used exclusively for DNA methylation analysis ‒ ULMS only.

| **Sample ID** | | **Assay Performed** | **Tumor Category** | **Institution** | **Collection Years** | **Storage Duration** |
| --- | --- | --- | --- | --- | --- | --- |
| EB16877 | EB16885 | Methylation | ULMS | ICESP | 2012–2020 | 4–12 years |
| EB16878 | EB16886 |  |  |  |  |  |
| EB16879 | EB16887 |  |  |  |  |  |
| EB16880 | EB16888 |  |  |  |  |  |
| EB16881 | EB16889 |  |  |  |  |  |
| EB16882 | EB16890 |  |  |  |  |  |
| EB16883 | EB16891 |  |  |  |  |  |
| EB16884 |  |  |  |  |  |  |

**Supplementary Table 3** Sample distribution across assays.

| **Assay** | **Sample type** | **Groups included** | **n (samples)** |
| --- | --- | --- | --- |
| DNA methylation (Illumina EPIC array) | FFPE | ULMS | 15^a^ |
|  | External dataset (GEO) | Myometrium (MM) | 10 |
| Gene expression (qRT-PCR) | Fresh-frozen | ULMS (n = 3), US (n = 6), LMU (n = 3), MM (n = 3) | 15 |
| Sanger sequencing | Fresh-frozen | Same samples as above | 15^b^ |
| Cell lines | In vitro | PCS-460-011 (LMU), SK-UT-1 (ULMS) | 2 |

^a^ Sixteen FFPE samples were initially processed, of which 15 passed quality control.

^b^ Performed according to DNA quality and target regions.

**Supplementary Figure 1** Panels A and B show density plots of β-values before and after normalization, respectively. In panel A (before), samples display broader and more heterogeneous β-value distributions, while in panel B (after) the distributions overlap more closely following BMIQ normalization and ComBat batch correction. Panels C and D present MDS plots based on the 1,000 most variable CpG sites before and after batch correction. Before correction (panel C), samples cluster strongly according to dataset origin, indicating the presence of batch effects; after correction (panel D), this separation is reduced, although some residual technical variation remains. Panels E and F show hierarchical clustering dendrograms before and after batch correction. In panel E (before), clustering is predominantly driven by technical batches, whereas in panel F (after), samples show more coherent organization and reduced batch-driven grouping. Together, these analyses demonstrate that BMIQ normalization followed by ComBat adjustment decreases technical variability and improves the comparability of internal samples with external controls.


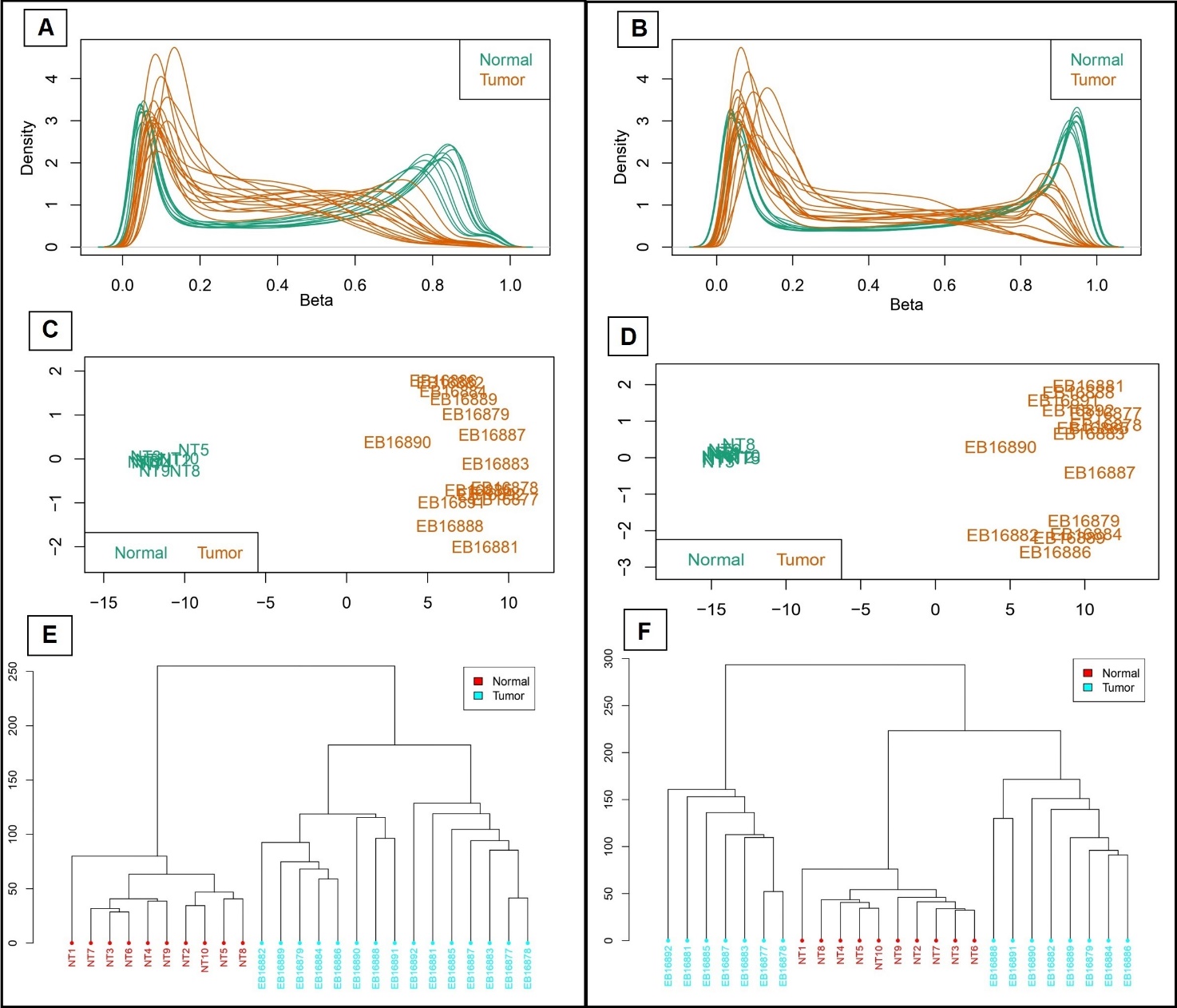

Supplement: Supplementary file 1 [file mmc1.docx]
